# Modeling the Dynamics of Dengue Transmission with Awareness and Optimal Control Analysis

**DOI:** 10.1371/journal.pone.0322702

**Published:** 2025-05-22

**Authors:** Faishal F. Herdicho, F. Fatmawati, Cicik Alfiniyah, Farah P. Fajrin, Ebenezer Bonyah, Muhammad A. Rois, Olumuyiwa J. Peter

**Affiliations:** 1 Department of Mathematics, Faculty of Science and Technology, Universitas Airlangga, Surabaya, Indonesia; 2 Department of Mathematics Education, Faculty of Applied Sciences and Mathematics Education, Akenten Appiah Menka University of Skills Training and Entrepreneurial Development, Kumasi, Ghana; 3 Department of Mathematics and Applied Mathematics, University of Johannesburg, Johannesburg, South Africa; 4 Department of Mathematics, Universitas Islam Negeri Salatiga, Salatiga, Indonesia; 5 Department of Mathematics, Saveetha School of Engineering, SIMATS, Saveetha University, Chennai, Tamil Nadu, 602105, India; 6 Department of Mathematical and Computer Sciences, University of Medical Sciences, Ondo City, Ondo State, Nigeria; 7 Department of Epidemiology and Biostatistics, School of Public Health, University of Medical Sciences, Ondo City, Ondo State, Nigeria; Parahyangan Catholic University: Universitas Katolik Parahyangan, INDONESIA

## Abstract

Dengue fever is a vector-borne disease which is transmitted by the bites of mosquitoes infected with dengue viruses. This disease is spread around the world and still as a global health problem. In this work, we formulate the dengue model by considering the population of aware humans. The real data of dengue infection in East Java, Indonesia is employed to estimate the parameters of the dengue model. The estimation of parameters is done by using monthly cumulative data on humans infected dengue that recorded at East Java Health Office on 2018-2020. We then analyze the stability of the equilibria of the model. The analysis exhibits that the disease-free equilibrium is locally and globally asymptotically stable when the basic reproduction number is less than one. We utilize the Lyapunov function approach to guarantee that the endemic equilibrium is globally asymptotically stable whenever the reproduction number is greater than one. Furthermore, this work examines the effectiveness of various dengue control strategies, including vector control, awareness program, and prevention. Cost-effectiveness evaluation has shown that the combination of vector control, awareness programs, and awareness prevention is the most effective intervention to reduce the dengue fever in the community.

## 1 Introduction

Dengue fever is an infectious disease caused by the dengue virus which is transmitted through one of the Aedes mosquito species, including Aedes aegypti, Aedes albopictus, and Aedes scutellaria [1]. Dengue virus is a group of Arthropod-Borne virus, genus flavivirus, family Flaviviridae. This virus consists of four different serotypes namely DEN-1, DEN-2, DEN-3, and DEN-4 [1]. After the bite of the mosquito infected, the patient will experience an incubation period of 4 to 10 days before symptoms appear. The symptoms experienced by the patient are forcing a high fever continuously for two until seven days, red spots appear on the skin, diarrhea, nausea, vomiting, dizziness, and a significant decrease in platelets. A patient will be called entering the critical phase if about three until seven days the fever which was initially 40°C will drop below 38°C [1]. Prevention and control of the spread of dengue fever relies heavily on vector control management and awareness campaigns. To date, there is no specific treatment for dengue fever. Early detection and access to appropriate medical care is an effective way to reduce the death rate due to severe dengue fever [1].

Dengue fever appears in tropical and sub-tropical areas. Climatic factors such as rainfall, temperature, humidity, and time of the rain are very influential on the breeding of Aedes mosquitoes. This causes a lot of standing water, so mosquitoes breed [2]. More than 3.9 billion people in more than 129 countries are at risk of contracting dengue fever, with an estimated 96 million symptomatic cases and around 40,000 deaths each year [3]. Indonesia is a tropical country that has a high number of dengue cases every year. One factor in the number of dengue cases in Indonesia is the high population density. Three provinces on the island of Java, namely West Java, East Java, and Central Java, have the highest population in Indonesia [4] and are the largest contributor to dengue cases during 2016–2020 [5]. East Java is the province with the second most populous population and has the second highest number of dengue cases in Indonesia during 2016–2020 [6].

In recent decades, mathematical modeling has played a fundamental role in understanding the dynamics of the spread of dengue fever. The dynamics of dengue fever transmission can be formulated using one of the epidemiological models called the compartment model which has been proposed and developed by many authors [7–10]. An epidemic outbreak typically begins with a single infected individual, known as patient zero, who first contracts the virus [11]. The Susceptible-Infectious-Recovered (SIR) model [11] is commonly used to analyze this transmission dynamic. Despite its simplicity, the SIR model is widely recognized in epidemiology because it effectively predicts a crucial concept: the epidemic threshold. This threshold distinguishes two possible epidemic outcomes a disease-free state and a scenario where a significant portion of the population becomes infected [12]. While more complex models exist, most are built upon the foundational principles of the SIR model, which accurately captures the fundamental dynamics of disease spread [13].

A number of studies have attempted a real data on the dengue model to predict its spread. The authors in [14] investigated the impact of the imperfect vaccine to control dengue virus transmission in Pakistan using a mathematical model. The authors in [15] developed a dengue model with saturated incidence rate to analyze the transmission of dengue in Bangladesh. The work of [16] proposed the dynamics of single and two-serotype dengue model with vaccination in Kupang city, Indonesia. The fractal-fractional Atangana-Baleanu model of dengue with hospitalization in East Java, Indonesia has been investigated by [17]. The study of [18] has devoted the integer-order model by considering temperature for the dengue outbreak in Malaysia along with the fractional-order. The authors in [19] have developed the time-dependent four-age structure model for dengue transmission in Bandung, Indonesia. In [20], the authors have devoted the time-varying effective reproduction number of the epidemic model to describe the spread of dengue fever in Palu City, Indonesia.

The concept of the optimal control theory has been studied extensively by researchers to determine optimal intervention strategies to prevent and reduce the number of human populations infected with dengue, see the literature [21–24]. For instance, the work in [21] has utilized optimal control strategies for dengue model with hospitalization to examine the effect of prevention and insecticides in reducing the spread of dengue fever in East Java Province, Indonesia. The extended control of dengue model with partial immune and asymptomatic individuals has been considered in [22]. The impact of vaccination, vector control, and media campaigns with seasonally varying mosquito populations on dengue transmission dynamics has been formulated in [23]. The study in [24] implemented the optimal control on the dengue fever model by considering asymptomatic, isolated, and alert compartments in the human population.

Individual awareness and willingness to take effective preventive measures to reduce disease transmission are important aspects in implementing dengue control strategies. A number of mathematical models to investigate the impact of individual awareness on the disease transmission dynamics have been discussed in [25–27]. The dynamical model of dengue fever spread taking into account individual awareness has been proposed in [28]. They considered the impact of the media campaign, case detection, and the hospital capacity to control dengue transmission in Jakarta, Indonesia through the novel mathematical model. In this work, we investigate the dengue model with awareness using dengue fever data in East Java, Indonesia. In the study of [28], it is assumed that the recruitment rate of the human population only enters the susceptible population. In our proposed model, the recruitment rate in a susceptible human population is separated into a portion entering the unaware population and a portion entering the aware population. Next, we extend the dengue model by incorporating three time-dependent optimal control interventions. The non-autonomous model is further analyzed using the well-known Pontryagin maximum principle.

The presentation of the work is structured as follows. Sect 2 is devoted to the dengue model formulation and the investigation of the basic properties of the model. Parameter estimation is explored in Sect 3. The local and global stability analysis is examined in Sect 4. Sect 5 contains a presentation on how the model parameters influence changes in disease outbreaks. In Sects 6 and 7, the proposed dengue model is extended to the optimal control problem and the simulation of the optimal control is demonstrated, respectively. The cost effectiveness analysis is presented in Sect 8. The study is ended with a conclusion in Sect 9.

## 2 Model formulation

The classical SIR model, which divides the population into susceptible (*S*), infected (*I*), and recovered (*R*) compartments, serves as a foundational framework for understanding disease dynamics. However, to better capture the complexities of dengue fever transmission, the model needs to be extended to account for additional factors such as hospitalization and individual awareness. A key transformation involves introducing a hospitalized (*P*) compartment, where infected individuals who seek medical treatment or are notified as infectious are separated from the general infected population. This modification allows for a more accurate representation of disease progression, as it accounts for individuals who receive medical care and do not contribute to further transmission. By incorporating awareness dynamics, the model also distinguishes between individuals who are informed about dengue prevention measures and those who are not, providing a more realistic approach to disease modeling.

In this section, we present the dengue fever model by incorporate the unaware and aware human population. The total mosquito population (*N*_*m*_) is separated into susceptible (*S*_*m*_) and infectious (*I*_*m*_) mosquitoes. The total human population (*N*_*h*_) is divided into unaware susceptible (*S*_*hu*_), aware susceptible (*S*_*ha*_), infectious (*I*_*h*_), hospitalized and/or notified infectious (*P*_*h*_), and recovered (*R*_*h*_).

**Fig 1:**
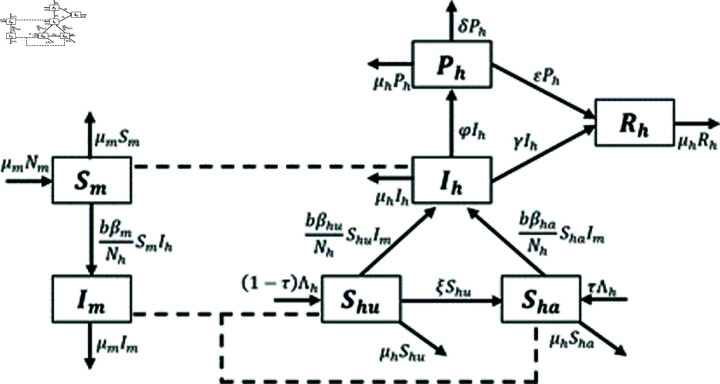
Transmission diagram of dengue.

The following assumptions is needed to explain the model construction. We assume that the total mosquito population (*N*_*m*_) is constant. The recruitment rate of the mosquito population is assumed to be equal to the natural mortality rate of mosquitoes. Mosquitoes, once infected, will remain infected for life. Each mosquito bite has an equal chance of spreading the virus to people in a susceptible population. In human populations, the recruitment rate is assumed to be constant, with some entering the unaware susceptible human population and some entering the aware susceptible human population. The human population in compartment *I*_*h*_ is assumed to be able to recover without having to enter compartment *P*_*h*_. All human populations included in compartment *P*_*h*_ are assumed to be 100% protected so that they do not contribute to the spread of dengue disease. The description of the model parameters are given in Table 1. The transmission diagram to devote all the interactions between the above compartments is presented in Fig 1.

**Table 1:** Definition of model parameters.

Notation	Notation
$\mu_{m}$	Natural death rate of mosquito
$\Lambda_{h}$	Recruitment rate of human
$\mu_{h}$	Natural death rate of human
*b*	Biting rate
$\beta_{m}$	Transmission probability from human to mosquito
$\beta_{ha}$	Transmission probability from aware human to mosquito
$\beta_{hu}$	Transmission probability from unaware human to mosquito
δ	Disease-induced death rate for human
φ	Rate of hospitalized and/or notified for human
ε	Recovery rate of the hospitalized and/or notified human
γ	Natural recovery rate of the human population
τ	Proportion of individuals who are naturally aware
ξ	Change rate from *S*_*hu*_ to *S*_*ha*_

With the above discussion, we display a system of nonlinear differential equations depicting the dynamics of host-vector dengue as follows:


$$\frac{dS_{m}}{dt}=\mu_{m}N_{m}-\frac{b\beta_{m}}{N_{h}}S_{m}I_{h}-\mu_{m}S_{m},$$

$$\frac{dI_{m}}{dt}=\frac{b\beta_{m}}{N_{h}}S_{m}I_{h}-\mu_{m}I_{m},$$

$$\frac{dS_{hu}}{dt}=\left(1-\tau \right)\Lambda_{h}-\frac{b\beta_{hu}}{N_{h}}S_{hu}I_{m}-\left(\mu_{h}+\xi \right)S_{hu},$$

$$\frac{dS_{ha}}{dt}=\tau \Lambda_{h}+\xi S_{hu}-\frac{b\beta_{ha}}{N_{h}}S_{ha}I_{m}-\mu_{h}S_{ha},$$ (1)

$$\frac{dI_{h}}{dt}=\frac{b\beta_{hu}}{N_{h}}S_{hu}I_{m}+\frac{b\beta_{ha}}{N_{h}}S_{ha}I_{m}-\left(\mu_{h}+\gamma +\varphi \right)I_{h},$$

$$\frac{dP_{h}}{dt}=\varphi I_{h}-\left(\mu_{h}+\varepsilon +\delta \right)P_{h},$$

$$\frac{dR_{h}}{dt}=\gamma I_{h}+\varepsilon P_{h}-\mu_{h}R_{h}.$$

The total mosquito populations $\left(N_{m}\right)$ and human populations $\left(N_{h}\right)$ respectively can be expressed as $N_{m}=S_{m}+I_{m}$ and $N_{h}=S_{hu}+S_{ha}+I_{h}+P_{h}+R_{h}$. The model Eq 1 subject to the initial conditions by $S_{m}\left(0\right)$, $S_{hu}\left(0\right)$, $S_{ha}\left(0\right)>0$ and $I_{m}\left(0\right)$, $I_{h}\left(0\right)$, $P_{h}\left(0\right)$, $R_{h}\left(0\right)\geq 0$, with the solutions remain non-negative for all time *t*>0 and defined in closed set Ω (positively invariant) given as

$$\Omega =\Omega_{m}\cup \Omega_{h}\subset {\mathbb{R}}_{+}^{2}\times {\mathbb{R}}_{+}^{5},$$ (2)

with


$$\Omega_{m}=\left\{\left(S_{m}\left(t\right),I_{m}\left(t\right)\right)\in {\mathbb{R}}_{+}^{2}\;:N_{m}=K\;|\;K\;is\;constant\;from\;S_{m}\left(0\right)+I_{m}\left(0\right)\right\},$$

$$\Omega_{h}=\left\{\left(S_{hu}\left(t\right),S_{ha}\left(t\right),I_{h}\left(t\right),P_{h}\left(t\right),R_{h}\left(t\right)\right)\in {\mathbb{R}}_{+}^{5}\;:N_{h}\leq \frac{\Lambda_{h}}{\mu_{h}}\right\}.$$

Next, we provide the positivity of solving the system Eq 1 according to the following theorem.

**Theorem 1**. *Let $S_{m}\left(0\right)$, $I_{m}\left(0\right)$, $S_{hu}\left(0\right)$, $S_{ha}\left(0\right)$, $I_{h}\left(0\right)$, $P_{h}\left(0\right)$, and $R_{h}\left(0\right)$ be the initial conditions of the system. If $S_{m}\left(0\right)\geq 0$, $I_{m}\left(0\right)\geq 0$, $S_{hu}\left(0\right)\geq 0$, $S_{ha}\left(0\right)\geq 0$, $I_{h}\left(0\right)\geq 0$, $P_{h}\left(0\right)\geq 0$, and $R_{h}\left(0\right)\geq 0$, then all solutions are nonnegative for every t≥0.*

*Proof:*

1. Carry out the first equation of the system Eq 1 as follows:
  $$\frac{dS_{m}\left(t\right)}{dt}=\mu_{m}N_{m}-\frac{b\beta_{m}}{N_{h}\left(t\right)}S_{m}\left(t\right)I_{h}\left(t\right)-\mu_{m}S_{m}\left(t\right).$$
  Let $\eta \left(t\right)=\frac{b\beta_{m}I_{h}\left(t\right)}{N_{h}\left(t\right)}$, so it is obtained as follows:
  $$\frac{d\left(e^{\mu_{m}t+\int_{0}^{t}\eta \left(s\right)\;ds}S_{m}\left(t\right)\right)}{dt}=\mu_{m}N_{m}e^{\mu_{m}t+\int_{0}^{t}\eta \left(s\right)\;ds}.$$ (3)
  Then the homogeneous solution is obtained
  $$\frac{d\left(e^{\mu_{m}t+\int_{0}^{t}\eta \left(s\right)\;ds}S_{m}\left(t\right)\right)}{dt}=0$$
  $$⟺S_{m}\left(t\right)=k\left(t\right)e^{-\mu_{m}t-\int_{0}^{t}\eta \left(s\right)\;ds}.$$ (4)
  Thus, let’s assume that the solution is non-homogeneous, substituted Eq 4 into Eq 3 we get
  $$\frac{dk\left(t\right)}{dt}=\mu_{m}N_{m}e^{\mu_{m}t+\int_{0}^{t}\eta \left(s\right)\;ds}$$
  $$⟺k\left(t\right)=\int_{0}^{t}\mu_{m}N_{m}e^{\mu_{m}w+\int_{0}^{w}\eta \left(x\right)\;dx}dw+K.$$ (5)
  Substituted Eq 5 into Eq 4 with the initial condition *S*_*m*_(0) at *t* = 0, we get
  $$S_{m}\left(t\right)=\int_{0}^{t}\mu_{m}N_{m}e^{\mu_{m}w+\int_{0}^{w}\eta \left(x\right)\;dx}dw\times \;e^{-\mu_{m}t-\int_{0}^{t}\eta \left(s\right)\;ds}+S_{m}\left(0\right)e^{-\mu_{m}t-\int_{0}^{t}\eta \left(s\right)\;ds}.$$
  So *S*_*m*_(*t*) is nonnegative for t≥0. Then using the same steps, we can prove the *S*_*hu*_(*t*) and $S_{ha}\left(t\right)$ is also nonnegative for t≥0.
2. Take the second equation of the system Eq 1 as follows:
  $$\frac{dI_{m}\left(t\right)}{dt}=\frac{b\beta_{m}}{N_{h}\left(t\right)}S_{m}\left(t\right)I_{h}\left(t\right)-\mu_{m}I_{m}\left(t\right)$$
  $$⟺\frac{dI_{m}\left(t\right)}{dt}\geq -\mu_{m}I_{m}\left(t\right)$$
  with the initial condition *I*_*m*_(0) at *t* = 0 we get
  $$I_{m}\left(t\right)\geq I_{m}\left(0\right)e^{-\mu_{m}t}$$
  So *I*_*m*_(*t*) is nonnegative for t≥0. Then using the same steps, we can prove the *I*_*h*_(*t*), $P_{h}\left(t\right)$, and $R_{h}\left(t\right)$ is also nonnegative for t≥0.

◻

## 3 Parameter estimation

In this section, the parameter estimation will be carried out on the model Eq 1. We obtained some of the parameter values from literature and others were estimated using the least-squares method. The data used is the cumulative data of dengue fever cases every month in January 2018 - December 2020 [6]. Then the average of human life expectancy in East Java from 2018 to 2020 is 71.15 years [29] with the average of total population in East Java from 2018 to 2020 is 39,955,059 people [30] so the calculation of parameters $\mu_{h}$ and $\Lambda_{h}$ as follows:


$$\mu_{h}=\frac{1}{Life\;Expectancy}=\frac{1}{71,15}=\frac{0.0140548138}{year}=1.172\;\times \frac{10^{-3}}{month},$$

$$\Lambda_{h}=\mu_{h}\times Total\;Population=561,560\frac{population}{year}=46,796\frac{population}{month}.$$

The remaining of the parameters in model Eq 1 is estimated with fulfill the condition $\beta_{hu}>\beta_{ha}$ and the goal is to minimize the objective function,


$${min}_{\tau ,b,\beta_{hu},\beta_{ha},\beta_{m},\mu_{m},\xi ,\;\varphi ,\gamma ,\varepsilon ,\delta }\sum_{i=0}^{t_{f}}{\left(P_{h_{i}}-Data_{i}\right)}^{2}\;,$$

where *t*_*f*_ is the end time of the cumulative dengue fever cases $Data_{i}$ $\left(i=0,1,2,\ldots ,t_{f}\right)$ and the cumulative numerical solutions of notified or hospitalized infected humans from model $P_{h_{i}}$ $\left(i=0,1,2,\ldots ,t_{f}\right)$. Next, we set the initial population is

$\left(S_{m_{0}};I_{m_{0}};S_{{hu}_{0}};S_{{ha}_{0}};I_{h_{0}};P_{h_{0}};R_{h_{0}}\right)=$

(158,087,600;300;26,347,933;13,173,967;2000;1106;100).

Thus, the initial parameters value for estimation is

$\left(\Lambda_{h_{0}};\tau_{0};b_{0};\beta_{hu_{0}};\beta_{ha_{0}};\beta_{m_{0}};{\mu_{m}}_{0};\xi_{0};\varphi_{0};\gamma_{0};\varepsilon_{0};\delta_{0}\right)=$

(0.4;0.6;0.7;0.6;0.7;9;0.5;0.07;0.04;0.06;0.005),

with lower bound and upper bound parameters value respectively is

$\left(10^{-10};10^{-10};\;10^{-10};10^{-10};10^{-10};1;10^{-10};10^{-10};\;10^{-10};10^{-10};10^{-10}\right)\text{and}$

(1;1;1;1;1;30;1;1;1;1;1).

**Fig 2:**
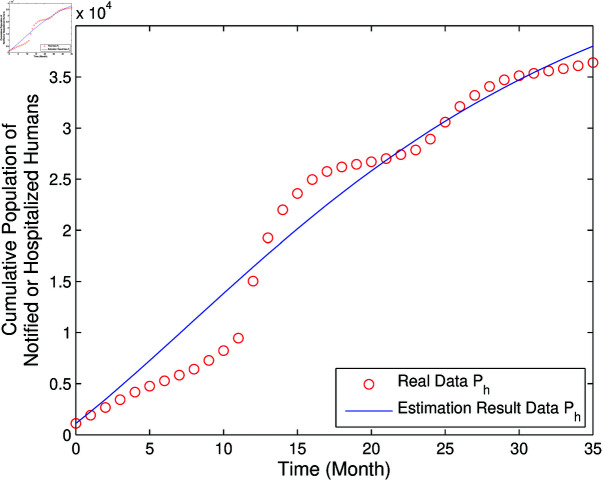
Comparison of the real data and estimation result of *P*_*h*_.

The result of estimation and parameter values are summarized in Fig 2 and Table 2.

**Table 2:** Fitted and estimated values of the parameters.

Parameters	Value	Source	Parameters	Value	Source
$\mu_{m}$	8.4277	Fitted	δ	0.0106	Fitted
$\Lambda_{h}$	46796	Estimated	φ	0.0702	Fitted
$\mu_{h}$	1.172	Estimated	ε	0.0655	Fitted
*b*	0.6443	Fitted	γ	0.0614	Fitted
$\beta_{m}$	0.7445	Fitted	τ	0.5562	Fitted
$\beta_{ha}$	0.4828	Fitted	ξ	0.2315	Fitted
$\beta_{hu}$	0.9961	Fitted			

Looking at Fig 2, it is apparent that the real data of the number of people who were hospitalized due to dengue fever with the calculation results had the same tendency.

## 4 Stability analysis

Equilibrium state is a state when the change in the population of each model variable over time is zero. Based on this statement, the mathematical model of dengue fever transmission satisfies a state of equilibrium when

$$\frac{dS_{m}}{dt}=\frac{dI_{m}}{dt}=\frac{dS_{hu}}{dt}=\frac{dS_{ha}}{dt}=\frac{dI_{h}}{dt}=\frac{dP_{h}}{dt}=\frac{dR_{h}}{dt}=0.$$ (6)

From Eq 6, we obtained two equilibrium points, namely disease-free and endemic equilibrium points. The disease-free equilibrium is a condition when there is no spread of disease $\left(I_{m}=I_{h}=P_{h}=0\right)$. While the endemic equilibrium point is the condition when the disease spreads $\left(I_{m}\neq 0,\;I_{h}\neq 0,P_{h}\neq 0\right)$. The disease-free equilibrium of the dengue spread model is provided by


$$E_{0}=\left(S_{m}^{0},\;I_{m}^{0},\;S_{hu}^{0},\;S_{ha}^{0},\;I_{h}^{0},\;P_{h}^{0},\;R_{h}^{0}\right)=\left(N_{m},\;0,\frac{\left(1-\tau \right)\Lambda_{h}}{\mu_{h}+\xi },\frac{\Lambda_{h}\left(\tau \mu_{h}+\xi \right)}{\mu_{h}\left(\mu_{h}+\xi \right)},\;0,\;0,\;0\right).$$

Next, we determined the basic reproduction number (*R*_0_). Using the Next Generation Matrix method [31], we have the the basic reproduction number as follow

$$R_{0}=\sqrt{\frac{b^{2}N_{m}\beta_{m}\mu_{h}\left(\beta_{hu}\left(1-\tau \right)\mu_{h}+\beta_{ha}\left(\tau \mu_{h}+\xi \right)\right)}{\mu_{m}\Lambda_{h}\left(\mu_{h}+\xi \right)\left(\mu_{h}+\gamma +\varphi \right)}}.$$ (7)

### 4.1 The local stability of the disease-free equilibrium

The local stability of the the disease-free equilibrium (*E*_0_) is yielded by substituting the *E*_0_ into the Jacobian matrix as follows:


$$J_{E_{0}}=\left(\begin{array}{ccccccc} -\mu_{m} & 0 & 0 & 0 & -\frac{b\beta_{m}N_{m}\mu_{h}}{\Lambda_{h}} & 0 & 0\\ 0 & -\mu_{m} & 0 & 0 & \frac{b\beta_{m}N_{m}\mu_{h}}{\Lambda_{h}} & 0 & 0\\ 0 & -\frac{b\beta_{hu}\left(1-\tau \right)\mu_{h}}{\mu_{h}+\xi } & -m_{1} & 0 & 0 & 0 & 0\\ 0 & -\frac{b\beta_{ha}\left(\tau \mu_{h}+\xi \right)}{\mu_{h}+\xi } & \xi & -\mu_{h} & 0 & 0 & 0\\ 0 & \frac{b\beta_{hu}\left(1-\tau \right)\mu_{h}+b\beta_{ha}\left(\tau \mu_{h}+\xi \right)}{\mu_{h}+\xi } & 0 & 0 & -m_{2} & 0 & 0\\ 0 & 0 & 0 & 0 & \varphi & -m_{3} & 0\\ 0 & 0 & 0 & 0 & \gamma & \varepsilon & -\mu_{h} \end{array}\right),$$

where


$$m_{1}=\mu_{h}+\xi ,\;m_{2}=\mu_{h}+\gamma +\varphi ,\;m_{3}=\left(\mu_{h}+\varepsilon +\delta \right).$$

From the matrix $J_{E_{0}}$, we will look for the characteristic equation with $det\left(\lambda I-J_{E_{0}}\right)=0\;$, so we get:

$${\left(\lambda +\mu_{h}\right)}^{2}\left(\lambda +\mu_{m}\right)\left(\lambda +\mu_{h}+\varepsilon +\delta \right)\left(\lambda +\mu_{h}+\xi \right)\left(\lambda^{2}+\lambda a_{1}+a_{2}\right)=0$$ (8)

where


$$a_{1}=\mu_{m}+\mu_{h}+\gamma +\varphi ,$$

$$a_{2}=\mu_{m}\left(\mu_{h}+\gamma +\varphi \right)\left[1-R_{0}^{2}\right].$$

From Eq 8, we obtain the eigenvalues $-\mu_{h},-\mu_{m},-(\mu_{h}+\varepsilon +\delta ),-(\mu_{h}+\xi )$ are obviously negative, while the remaining two eigenvalues are the roots of the following equation:

$$\lambda^{2}+\lambda a_{1}+a_{2}=0.$$ (9)

Using Routh-Hurwitz criterion, the characteristic Eq 9 will have roots with real parts are negative if and only if $a_{1},\;a_{2}>0$. From the description obtained:

1. It is clear that *a*_1_>0 because all parameters are positive.
2. The coefficient $a_{2}>0\Leftrightarrow R_{0}^{2}<1\Leftrightarrow R_{0}<1$.

Hence, all roots of Eq 9 are negative real parts if *R*_0_<1. Therefore, it is proved that the disease-free equilibrium $\left(E_{0}\right)\;$will be locally asymptotically stable if *R*_0_<1 and unstable if *R*_0_>1. The foregoing discussion could be summarized in the following theorem.

**Theorem 2.** *The the disease-free equilibrium $\left(E_{0}\right)$ of the system* Eq 1 *is locally asymptotically stable in region of interest* 𝛺 if *R*_*0*_*<1 and unstable if* *R*_*0*_*>1.*

### 4.2 The global stability of the disease-free equilibrium

The global stability of the disease-free equilibrium is examined using the method described by Castillo-Chavez *et al*. in [32]. Let $X={\left(S_{m},S_{hu},S_{ha},R_{h}\right)}^{T}\in {\mathbb{R}}^{4}$ and $Z={\left(I_{m},I_{h},\;P_{h}\right)}^{T}\in {\mathbb{R}}^{3}$ and the system Eq 1 can be rewrite as follows:

$$\frac{dX}{dt}=F\left(X,Z\right)$$ (10)

$$\frac{dZ}{dt}=G(X,Z),G\left(X,0\right)=0,$$

where $E_{0}^{*}=\left(X^{*},0\right)$ represents the disease-free equilibrium of the system.

Based [32], the fixed point $E_{0}^{*}=\left(X^{*},0\right)$ is globally asymptotically stable provided that *R*_0_<1 and the two conditions bellow are fulfilled

(H1) For $\frac{dX}{dt}=F\left(X,0\right)$, ${\text{X}}^{*}$ is globally asymptotically stable,

(H2) $G\left(X,Z\right)=AZ-\hat{G}\left(X,Z\right)\geq 0$ for (X,Z)∈Ω,

where $A=D_{Z}G\left(X^{*},0\right)$ is the *M*-matrix (the off diagonal elements of *A* are non-negative).

From the system Eq 1, we can get form of Eq (10) as follows:


$$F\left(X,Z\right)=\left(\begin{array}{c} \mu_{m}N_{m}-\frac{b\beta_{m}}{N_{h}}S_{m}I_{h}-\mu_{m}S_{m}\\ \left(1-\tau \right)\Lambda_{h}-\frac{b\beta_{hu}}{N_{h}}S_{hu}I_{m}-\left(\mu_{h}+\xi \right)S_{hu}\\ \tau \Lambda_{h}+\xi S_{hu}-\frac{b\beta_{ha}}{N_{h}}S_{ha}I_{m}-\mu_{h}S_{ha}\\ \gamma I_{h}+\varepsilon P_{h}-\mu_{h}R_{h} \end{array}\right),$$

$$G\left(X,Z\right)=\left(\begin{array}{c} \frac{b\beta_{m}}{N_{h}}S_{m}I_{h}-\mu_{m}I_{m}\\ \frac{b\beta_{hu}}{N_{h}}S_{hu}I_{m}+\frac{b\beta_{ha}}{N_{h}}S_{ha}I_{m}-\left(\mu_{h}+\gamma +\varphi \right)I_{h}\\ \varphi I_{h}-\left(\mu_{h}+\varepsilon +\delta \right)P_{h} \end{array}\right).$$

Furthermore


$$A=\left(\begin{array}{ccc} -\mu_{m} & \frac{b\beta_{m}}{N_{h}^{0}}S_{m}^{0} & 0\\ \frac{b\beta_{hu}}{N_{h}^{0}}S_{hu}^{0}+\frac{b\beta_{ha}}{N_{h}^{0}}S_{ha}^{0} & -\left(\mu_{h}+\gamma +\varphi \right) & 0\\ 0 & \varphi & -\left(\mu_{h}+\varepsilon +\delta \right) \end{array}\right),$$

$$\hat{G}\left(X,Z\right)=\left(\begin{array}{c} b\beta_{m}I_{h}\frac{S_{m}^{0}}{N_{h}^{0}}\left(1-\frac{S_{m}N_{h}^{0}}{N_{h}S_{m}^{0}}\right)\\ b\beta_{hu}I_{m}\left(1-\frac{S_{hu}N_{h}^{0}}{N_{h}S_{hu}^{0}}\right)+b\beta_{ha}I_{m}\left(1-\frac{S_{ha}N_{h}^{0}}{N_{h}S_{ha}^{0}}\right)\\ 0 \end{array}\right),$$

$$F\left(X,0\right)=\left(\begin{array}{c} \mu_{m}N_{m}-\mu_{m}S_{m}\\ \left(1-\tau \right)\Lambda_{h}-\left(\mu_{h}+\xi \right)S_{hu}\\ \tau \Lambda_{h}+\xi S_{hu}-\mu_{h}S_{ha}\\ -\mu_{h}R_{h} \end{array}\right).$$

Solving $\frac{dX}{dt}=F\left(X,0\right)$, we obtain

$$\left(\begin{array}{c} S_{m}\left(t\right)\\ S_{hu}\left(t\right)\\ S_{ha}\left(t\right)\\ R_{h}\left(t\right) \end{array}\right)=\left(\begin{array}{c} N_{m}+\left(S_{m}\left(0\right)-N_{m}\right)e^{-\mu_{m}t}\\ \frac{\left(1-\tau \right)\Lambda_{h}}{\left(\mu_{h}+\xi \right)}+\left(S_{hu}\left(0\right)-\frac{\left(1-\tau \right)\Lambda_{h}}{\left(\mu_{h}+\xi \right)}\right)e^{-\left(\mu_{h}+\xi \right)t}\\ \frac{\tau \Lambda_{h}+\xi S_{hu}}{\mu_{h}}+\left(S_{ha}\left(0\right)-\frac{\tau \Lambda_{h}+\xi S_{hu}}{\mu_{h}}\right)e^{-\mu_{h}t}\\ R_{h}\left(0\right)e^{-\mu_{h}t} \end{array}\right).$$ (11)

Hence, from Eq (11), when Z=0, we obtain ${lim}_{t\to \infty }S_{m}\left(t\right)\;=N_{m}=S_{m}^{0}$, ${lim}_{t\to \infty }S_{hu}\left(t\right)\;=\frac{\left(1-\tau \right)\Lambda_{h}}{\left(\mu_{h}+\xi \right)}=S_{hu}^{0}$,$\;{lim}_{t\to \infty }S_{ha}\left(t\right)\;=\frac{\Lambda_{h}\left(\tau \mu_{h}+\xi \right)}{\mu_{h}\left(\mu_{h}+\xi \right)}=S_{ha}^{0}$, and ${lim}_{t\to \infty }R_{h}\left(t\right)\;=0=R_{h}^{0}$ ensuring the global asymptotic stability of the equilibrium point $X^{*}=\left(N_{m},\frac{\left(1-\tau \right)\Lambda_{h}}{\left(\mu_{h}+\xi \right)},\frac{\Lambda_{h}\left(\tau \mu_{h}+\xi \right)}{\mu_{h}\left(\mu_{h}+\xi \right)},0\right)$. Hence H1 is satisfied.

Next, it is clear that $S_{m}\leq N_{m}=S_{m}^{0}$ and by solving the third and fourth equations on model Eq 1, we get ${lim}_{t\to \infty }S_{hu}\left(t\right)\;\leq \frac{\left(1-\tau \right)\Lambda_{h}}{\left(\mu_{h}+\xi \right)}=S_{hu}^{0}$ and ${lim}_{t\to \infty }S_{ha}\left(t\right)\;\leq \frac{\Lambda_{h}\left(\tau \mu_{h}+\xi \right)}{\mu_{h}\left(\mu_{h}+\xi \right)}=S_{ha}^{0}$ such that $S_{m}\leq S_{m}^{0}$, $S_{hu}\leq S_{hu}^{0}$, and $S_{ha}\leq S_{ha}^{0}$. However, to have ${\hat{G}}_{1}\left(X,Z\right)\geq 0$ and ${\hat{G}}_{2}\left(X,Z\right)\geq 0$, some conditions are required. For example, we could let the total human population be at equilibrium level $\left(N_{h}=\frac{\Lambda_{h}}{\mu_{h}}=N_{h}^{0}\right)$ and this condition will be achieved when we assume to ignore the disease-induced death rate. This ensures that $1-\frac{S_{m}N_{h}^{0}}{N_{h}S_{m}^{0}}\geq 0$, $1-\frac{S_{hu}N_{h}^{0}}{N_{h}S_{hu}^{0}}\geq 0$, and $1-\frac{S_{ha}N_{h}^{0}}{N_{h}S_{ha}^{0}}\geq 0$ such that H2 is satisfied.

Therefore, because two conditions are fulfilled so the disease-free equilibrium point is globally asymptotically stable if we assume to ignore the disease-induced death rate. The foregoing discussion could be summarized in the following theorem.

**Theorem 3.** *Suppose that in system Eq* \eqref1 the disease-induced death rate is ignored (δ=0). If *R*_*0*_*<1, then the disease-free equilibrium point is globally asymptotically stable.*

### 4.3 Endemic equilibrium

The endemic equilibrium of the system Eq 1 can be determined by using the conditions of the force infection (κ), with

$$\kappa_{hu}=b\frac{\beta_{hu}I_{m}}{N_{h}},\;\;\;\;\;\;\;\;\kappa_{ha}=b\frac{\beta_{ha}I_{m}}{N_{h}},\;\;\;\;\;\;\;\;\kappa_{m}=b\frac{\beta_{m}I_{h}}{N_{h}}.$$ (12)

The endemic equilibrium is obtained as follows:


$$E^{*}=\left(S_{m}^{*},\;I_{m}^{*},\;S_{hu}^{*},\;S_{ha}^{*},\;I_{h}^{*},\;P_{h}^{*},\;R_{h}^{*}\right),$$

where


$$S_{m}^{*}=\frac{\mu_{m}N_{m}}{\kappa_{m}^{*}+\mu_{m}},$$

$$I_{m}^{*}=\frac{\kappa_{m}^{*}}{\mu_{m}}S_{m}^{*},$$

$$S_{hu}^{*}=\frac{\left(1-\tau \right)\Lambda_{h}}{\kappa_{hu}^{*}+\mu_{h}+\xi },$$

$$S_{ha}^{*}=\frac{\tau \Lambda_{h}+\xi S_{hu}^{*}}{\kappa_{ha}^{*}+\mu_{h}},$$

$$I_{h}^{*}=\frac{\kappa_{hu}^{*}S_{hu}^{*}+\kappa_{ha}^{*}S_{ha}^{*}}{\mu_{h}+\gamma +\varphi },$$

$$P_{h}^{*}=\frac{\varphi }{\mu_{h}+\varepsilon +\delta }I_{h}^{*},$$

$$R_{h}^{*}=\frac{\gamma I_{h}^{*}+\varepsilon P_{h}^{*}}{\mu_{h}}.$$

In this case, we ignore the disease-induced death rate (δ=0) to show the continuation of Theorem 3. We note that the disease-free equilibrium is globally asymptotically stable, which means that the existence of backward bifurcation will not occur when the disease-induced death rate is zero. Furthermore, when δ=0, we obtain $N_{h}^{*}=\frac{\Lambda_{h}}{\mu_{h}}$ and the force of infection at equilibrium conditions as follows:


$$\kappa_{hu}^{*}=\frac{b\beta_{hu}\mu_{m}N_{m}\kappa_{m}^{*}}{N_{h}^{*}\mu_{m}\left(\kappa_{m}^{*}+\mu_{m}\right)},$$

$$\kappa_{ha}^{*}=\frac{b\beta_{ha}\mu_{m}N_{m}\kappa_{m}^{*}}{N_{h}^{*}\mu_{m}\left(\kappa_{m}^{*}+\mu_{m}\right)},$$

and $\kappa_{m}^{*}$ is the roots of the following equation:


$$x{\kappa_{m}^{*}}^{2}+y\kappa_{m}^{*}+z=0,$$

with


$$x=N_{h}^{*}\left(\mu_{h}+\gamma +\varphi \right)\left(b\beta_{ha}\mu_{m}N_{m}+N_{h}^{*}\mu_{m}\mu_{h}\right)\left(b\beta_{hu}\mu_{m}N_{m}+N_{h}^{*}\mu_{m}\left(\mu_{h}+\xi \right)\right),$$

$$y=b\left(b\beta_{hu}N_{m}{N_{h}^{*}}^{2}\mu_{m}^{3}\mu_{h}\left(\mu_{h}+\gamma +\varphi \right)+b\beta_{ha}N_{m}{N_{h}^{*}}^{2}\mu_{m}^{3}\left(\mu_{h}+\xi \right)\left(\mu_{h}+\gamma +\varphi \right)\right)\left[1-R_{x}\right]\;\;+{N_{h}^{*}}^{3}\mu_{m}^{3}\mu_{h}\left(\mu_{h}+\xi \right)\left(\mu_{h}+\gamma +\varphi \right)\left[2-R_{0}^{2}\right],$$

$$z={N_{h}^{*}}^{3}\mu_{m}^{4}\mu_{h}\left(\mu_{h}+\xi \right)\left(\mu_{h}+\gamma +\varphi \right)\left[1-R_{0}^{2}\right],$$

with $R_{x}=\frac{b^{2}\beta_{m}\beta_{hu}\beta_{ha}N_{m}\mu_{h}^{2}}{\Lambda_{h}\mu_{m}\left(\mu_{h}+\gamma +\varphi \right)\left(\beta_{hu}\mu_{h}+\beta_{ha}\left(\mu_{h}+\xi \right)\right)}$.

Furthermore, using algebraic calculation, the relationship between $R_{0}^{2}\;$and *R*_*x*_ is given by


$$R_{0}^{2}-R_{x}=\frac{b^{2}N_{m}\beta_{m}\mu_{h}\left(\beta_{hu}^{2}\left(1-\tau \right)\mu_{h}^{2}+\beta_{ha}^{2}\left(\tau \mu_{h}+\xi \right)\left(\mu_{h}+\xi \right)+\beta_{hu}\beta_{ha}\left(1-\tau \right)\mu_{h}\xi \right)}{\Lambda_{h}\mu_{m}\left(\mu_{h}+\gamma +\varphi \right)\left(\mu_{h}+\xi \right)\left(\beta_{hu}\mu_{h}+\beta_{ha}\left(\mu_{h}+\xi \right)\right)}.$$

Because all the parameters are positive and 0≤τ≤1, so it is clear that $R_{0}^{2}-R_{x}>0\Leftrightarrow R_{x}<R_{0}^{2}.$

Suppose $z>0\Leftrightarrow R_{0}^{2}<1\Leftrightarrow R_{0}<1$. We have $R_{x}<R_{0}^{2}$, so $R_{x}<R_{0}^{2}<1\Leftrightarrow R_{x}<1$. It is clear that 1−*R*_*x*_>0 and $2-R_{0}^{2}>0$. Thus, when *z*>0, then *y*>0 cause the model does not has two endemic equilibrium points in Ω. Hence, the backward bifurcation does not occur in the model when we assume to ignore the disease-induced death rate.

Therefore, we obtained the following results:

**Theorem 4.** *Suppose that in system* Eq 1 *the disease-induced death rate is ignored *(δ=0)*. Then the system* Eq 1 *has:*

1. *A unique endemic equilibrium that exist in *𝛺 *if z*<0 $\left(i.e.\;R_{0}>1\right)$.
2. *A unique endemic equilibrium that exist in *𝛺 *if y*<0 *and either z* = 0 $\left(i.e.\;R_{0}=1\right)$ *or y*^2^−4*xz* = 0.
3. *No endemic equilibrium otherwise.*

### 4.4 The global stability of endemic equilibrium

Suppose


$$\Omega_{0}=\left\{M\in \Omega \;:I_{m}=I_{h}=P_{h}=R_{h}=0\right\},$$

with $M=\left(S_{m}\left(t\right),\;I_{m}\left(t\right),S_{hu}\left(t\right),\;S_{ha}\left(t\right),\;I_{h}\left(t\right),P_{h}\left(t\right),\;R_{h}\left(t\right)\right)$ and $\Omega_{0}$ is defined as the stable manifold of non-endemic equilibrium $\left(E_{0}\right)$. The global stability of endemic equilibrium is provided in the following theorem.

**Theorem 5.** *The endemic equilibrium *$\left(E^{*}\right)$ *is globaly asymptotically stable in the interior of region *$𝛺\backslash {𝛺}_{0}$ *if R*_0_>1, *supposing that, in system* Eq 1, *the disease-induced death rate is ignored *(δ=0).

*Proof:* We use Lyapunov function $ℒ\;:\;𝛺\backslash {𝛺}_{0}\to \mathbb{R}$ defined as


$$ℒ=\frac{1}{2}{\left[\left(S_{m}-S_{m}^{*}\right)+\left(I_{m}-I_{m}^{*}\right)\right]}^{2}\;\;+\frac{1}{2}{\left[\left(S_{hu}-S_{hu}^{*}\right)+\left(S_{ha}-S_{ha}^{*}\right)+\left(I_{h}-I_{h}^{*}\right)+\left(P_{h}-P_{h}^{*}\right)+\left(R_{h}-R_{h}^{*}\right)\right]}^{2}.$$

The time derivative of ℒ is

$$\frac{dℒ}{dt}=\left[\left(S_{m}+I_{m}\right)-\left(S_{m}^{*}+I_{m}^{*}\right)\right]\frac{dN_{m}}{dt}\;\;+\left[\left(S_{hu}+S_{ha}+I_{h}+P_{h}+R_{h}\right)-\left(S_{hu}^{*}+S_{ha}^{*}+I_{h}^{*}+P_{h}^{*}+R_{h}^{*}\right)\right]\frac{dN_{h}}{dt}$$ (13)

$$=\left(N_{m}-N_{m}^{*}\right)\frac{dN_{m}}{dt}+\left(N_{h}-N_{h}^{*}\right)\frac{dN_{h}}{dt}$$

$$=\left(N_{m}-N_{m}^{*}\right)0+\left(N_{h}-N_{h}^{*}\right)\left(\Lambda_{h}-\mu_{h}N_{h}-\delta P_{h}\right)$$

$$=\left(N_{h}-N_{h}^{*}\right)\left(\Lambda_{h}-\mu_{h}N_{h}-\delta P_{h}\right).$$

In this case, we analyze the global stability by assuming the disease-induced death rate is ignored (δ=0). This approach aims to demonstrate that, under these conditions, global stability will be guaranteed, and no backward bifurcation will occur, consistent with the endemic equilibrium point previously obtained. Therefore, we have $N_{h}^{*}=\frac{\Lambda_{h}}{\mu_{h}}$, and Eq (13) is simplified to:


$$\frac{dℒ}{dt}=\left(N_{h}-\frac{\Lambda_{h}}{\mu_{h}}\right)\left(\Lambda_{h}-\mu_{h}N_{h}\right)$$

$$=-\frac{1}{\mu_{h}}{\left(\Lambda_{h}-\mu_{h}N_{h}\right)}^{2}$$

Therefore, $\frac{dℒ}{dt}\leq 0$ due to all the parameters are positive, with $\frac{dℒ}{dt}=0$ if only if $S_{hu}=S_{hu}^{*}$, $S_{ha}=S_{ha}^{*}$, $I_{h}=I_{h}^{*}$, $P_{h}=P_{h}^{*}$, and $R_{h}=R_{h}^{*}$. The endemic equilibrium point ${\text{E}}^{*}$ exists if only if *R*_0_>1 and the singleton set $\left\{E^{*}\right\}$ is the biggest compact invariant set in $\{\left(S_{m},\;I_{m},S_{hu},\;S_{ha},\;I_{h},P_{h},\;R_{h}\right)$ $\in \Omega \;:\frac{dℒ}{dt}=0\}$. According to LaSalle’s invariance principle [33], the endemic equilibrium ${\text{E}}^{*}$ globally asymptotically stable in the interior of region $\Omega \backslash \Omega_{0}$ if $R_{0}>1$. ◻

## 5 Sensitivity analysis parameter

Sensitivity analysis is used to establish the most influential parameter in the model [34]. In this case, a sensitivity index will be determined for each of the eleven parameters involved in the basic reproduction number (*R*_0_) of the mathematical model of the dengue fever transmission. The parameter sensitivity index (*e*_*p*_) is formulated as follows:


$$e_{p}=\left(\frac{\partial R_{0}}{\partial p}\right)\left(\frac{p}{R_{0}}\right)$$

where pis the parameter to be analyzed.

Using the parameter values in Table 2, the sensitivity index of the parameters model to *R*_0_ are presented in Table 3.

**Table 3:** Sensitivity index of parameters.

Parameter (*p*)	Sensitivity index	Parameter (*p*)	Sensitivity index
$\mu_{m}$	-0.5	$\beta_{hu}$	0.0023
$\Lambda_{h}$	-0.5	φ	-0.2644
$\mu_{h}$	0.4968	γ	-0.2312
*b*	1	τ	-0.0015
$\beta_{m}$	0.5	ξ	-0.0012
$\beta_{ha}$	0.4977		

A positive sensitivity index interprets that the greater of the parameter values will cause an increase in the *R*_0_. Conversely, a negative sensitivity index means that the greater of the parameter values will cause a decrease in the *R*_0_. Based on Table 3, the most influential and controllable parameters are parameters *b*, $\beta_{m}$, $\mu_{m}$, and $\beta_{ha}$. Next, we will simulation the sensitivity of parameters *b*, $\beta_{m}$, $\mu_{m}$, and $\beta_{ha}$ to *R*_0_ contour plots.

**Fig 3:**
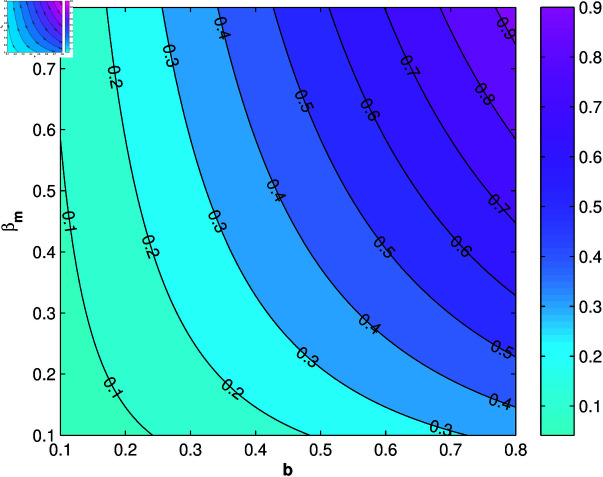
Contour plot of *R*_0_ due to change in $b-\beta_{m}$ values.

**Fig 4:**
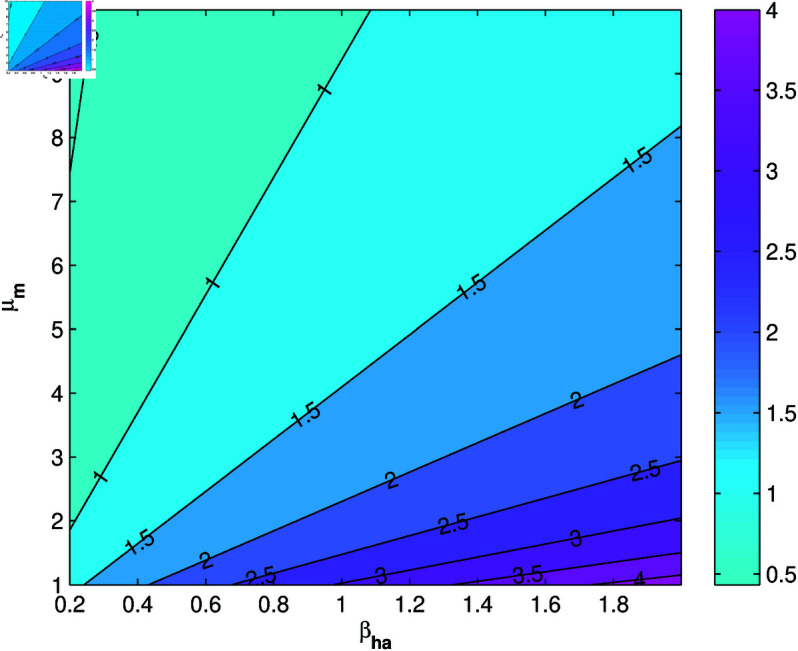
Contour plot of *R*_0_ due to change in $\beta_{ha}-\mu_{m}$ values.

**Fig 5:**
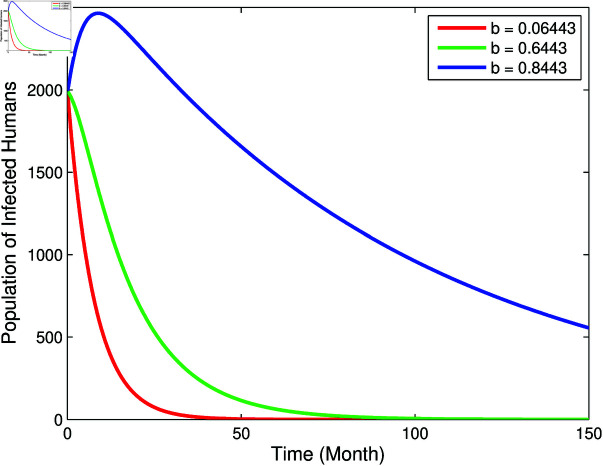
Graph of the effect of parameter b to population *I*_*h*_.

**Fig 6:**
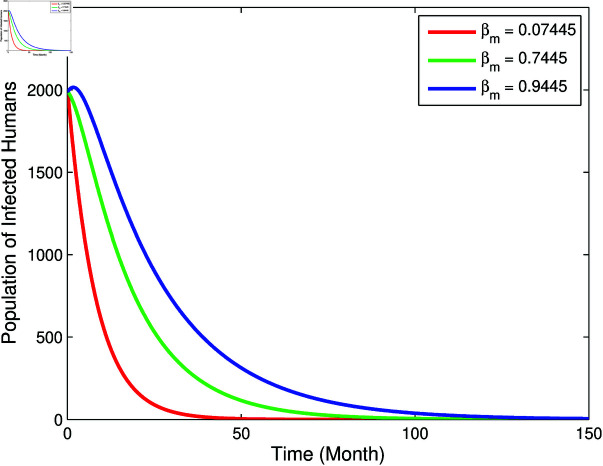
Graph of the effect of parameter $\beta_{m}$ to population *I*_*h*_.

**Fig 7:**
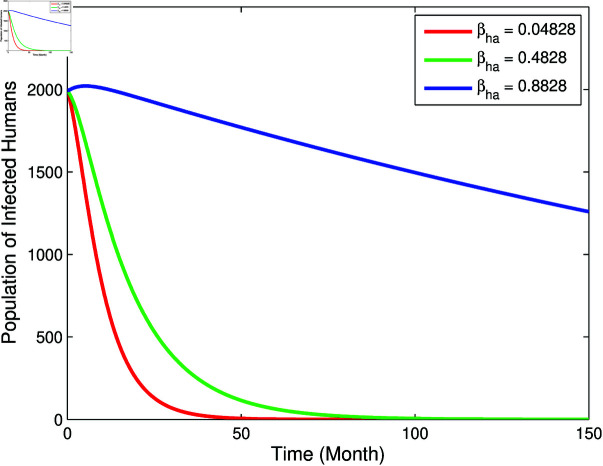
Graph of the effect of parameter $\beta_{ha}$ to population *I*_*h*_.

**Fig 8:**
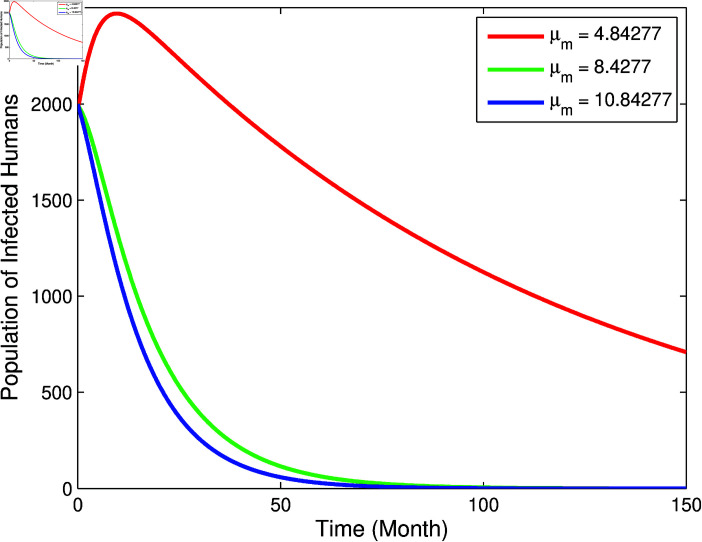
Graph of the effect of parameter $\mu_{m}$ to population *I*_*h*_.

From Figs 3 and 4, it can be seen that *b*, $\beta_{m}$, and $\beta_{ha}$ has a positive relation to *R*_0_, however $\mu_{m}$ has negative relation to *R*_0_, this corresponds to the sign of index sensitivity in Table 3. Furthermore, to ensure whether the parameters *b*, $\beta_{m}$, $\mu_{m}$, and $\beta_{ha}$ really has an effect, a simulation of changes in parameter values will be carried out on the population *I*_*h*_ with the results as follows:

From Figs 5–7, it can be seen that the greater value of *b*, $\beta_{m}$, and $\beta_{ha}$ causes the infected human population to be greater. While in Fig 8, the smallest value of $\mu_{m}$ cause the infected human population to be greater. These results are inline with the sensitivity index values in Table 3.

## 6 Optimal control problem

In this section, we perform an optimal control approach to examine the effects of control on dengue fever transmission dynamics. We extend model Eq 1 with three optimal control variables as follows: vector control $\left(u_{1}\right)$, awareness program $\left(u_{2}\right)$, and awareness prevention $\left(u_{3}\right)$ as follows:


$$\frac{dS_{m}}{dt}=\mu_{m}N_{m}-\frac{b\beta_{m}}{N_{h}}S_{m}I_{h}-\mu_{m}S_{m}-\sigma u_{1}S_{m},$$

$$\frac{dI_{m}}{dt}=\frac{b\beta_{m}}{N_{h}}S_{m}I_{h}-\mu_{m}I_{m}-\sigma u_{1}I_{m},$$

$$\frac{dS_{hu}}{dt}=\left(1-\tau \right)\Lambda_{h}-\frac{b\beta_{hu}}{N_{h}}S_{hu}I_{m}-\left(\mu_{h}+\xi \right)S_{hu}-\phi u_{2}S_{hu},$$

$$\frac{dS_{ha}}{dt}=\tau \Lambda_{h}+\xi S_{hu}-\left(1-u_{3}\right)\frac{b\beta_{ha}}{N_{h}}S_{ha}I_{m}-\mu_{h}S_{ha}+\phi u_{2}S_{hu},$$ (14)

$$\frac{dI_{h}}{dt}=\frac{b\beta_{hu}}{N_{h}}S_{hu}I_{m}+\left(1-u_{3}\right)\frac{b\beta_{ha}}{N_{h}}S_{ha}I_{m}-\left(\mu_{h}+\gamma +\varphi \right)I_{h},$$

$$\frac{dP_{h}}{dt}=\varphi I_{h}-\left(\mu_{h}+\varepsilon +\delta \right)P_{h},$$

$$\frac{dR_{h}}{dt}=\gamma I_{h}+\varepsilon P_{h}-\mu_{h}R_{h},$$

with σ and ϕ respectively is rate of implementation vector control and awareness program with $u_{i}\in \left[0,1\right]$, i=1,2,3. The purpose of the optimal control problem is to set the optimal values that minimize the following objective function:

$$𝒥=\int_{0}^{t_{f}}\left(A_{1}I_{m}+A_{2}I_{h}+A_{3}P_{h}+\frac{1}{2}A_{4}u_{1}^{2}+\frac{1}{2}A_{5}u_{2}^{2}+\frac{1}{2}A_{6}u_{3}^{2}\right)dt,$$ (15)

subject to system Eq 16, with *A*_1_, *A*_2_, *A*_3_, $A_{4},A_{5}$, and *A*_6_ are balancing coefficient for the controls and *t*_*f*_ is the final time. We employ a quadratic form in the control variables to exhibit the nonlinear costs of implementing the control strategies. Therefore, the quadratic form of the cost has been commonly utilized in various literature [35–37]. To solve this optimal control problem, we utilize Pontryagin Maximum Principle [38]. First, we establish the Hamiltonian function as follows:


$$ℋ=\left(A_{1}I_{m}+A_{2}I_{h}+A_{3}P_{h}+\frac{1}{2}A_{4}u_{1}^{2}+\frac{1}{2}A_{5}u_{2}^{2}+\frac{1}{2}A_{6}u_{3}^{2}\right)\;+\lambda_{1}\left(\mu_{m}N_{m}-\frac{b\beta_{m}}{N_{h}}S_{m}I_{h}-\mu_{m}S_{m}-\sigma u_{1}S_{m}\right)\;+\lambda_{2}\left(\frac{b\beta_{m}}{N_{h}}S_{m}I_{h}-\mu_{m}I_{m}-\sigma u_{1}I_{m}\right)\;+\lambda_{3}\left(\left(1-\tau \right)\Lambda_{h}-\frac{b\beta_{hu}}{N_{h}}S_{hu}I_{m}-\left(\mu_{h}+\xi \right)S_{hu}-\phi u_{2}S_{hu}\right)\;+\lambda_{4}\left(\tau \Lambda_{h}+\xi S_{hu}-\left(1-u_{3}\right)\frac{b\beta_{ha}}{N_{h}}S_{ha}I_{m}-\mu_{h}S_{ha}+\phi u_{2}S_{hu}\right)\;+\lambda_{5}\left(\frac{b\beta_{hu}}{N_{h}}S_{hu}I_{m}+\left(1-u_{3}\right)\frac{b\beta_{ha}}{N_{h}}S_{ha}I_{m}-\left(\mu_{h}+\gamma +\varphi \right)I_{h}\right)\;+\lambda_{6}\left(\varphi I_{h}-\left(\mu_{h}+\varepsilon +\delta \right)P_{h}\right)+\lambda_{7}\left(\gamma I_{h}+\varepsilon P_{h}-\mu_{h}R_{h}\right),$$

with $\lambda_{i}$, (i=1,2,3,…,6) are adjoint/co-state variables associated with each state variables. The co-state variables satisfies the following equations


$$\frac{d\lambda_{1}}{dt}=-\frac{\partial ℋ}{\partial S_{m}}=\left(\lambda_{1}-\lambda_{2}\right)\frac{b\beta_{m}I_{h}}{N_{h}}+\lambda_{1}\left(\mu_{m}+\sigma u_{1}\right),$$

$$\frac{d\lambda_{2}}{dt}=-\frac{\partial ℋ}{\partial I_{m}}=-A_{1}+\left(\lambda_{3}-\lambda_{5}\right)\frac{b\beta_{hu}S_{hu}}{N_{h}}+\left(\lambda_{4}-\lambda_{5}\right)\frac{\left(1-u_{3}\right)b\beta_{ha}S_{ha}}{N_{h}}+\lambda_{2}\left(\mu_{m}+\sigma u_{1}\right),$$

$$\frac{d\lambda_{3}}{dt}=-\frac{\partial ℋ}{\partial S_{hu}}=\left(\lambda_{2}-\lambda_{1}\right)\frac{b\beta_{m}S_{m}I_{h}}{N_{h}^{2}}+\left(\lambda_{3}-\lambda_{5}\right)\frac{b\beta_{hu}I_{m}N_{h}-b\beta_{hu}S_{hu}I_{m}}{N_{h}^{2}}\;\;\;\;\;\;+\left(\lambda_{5}-\lambda_{4}\right)\frac{\left(1-u_{3}\right)b\beta_{ha}S_{ha}I_{m}}{N_{h}^{2}}+\left(\lambda_{3}-\lambda_{4}\right)\left(\xi +\phi u_{2}\right)+\lambda_{3}\mu_{h},$$

$$\frac{d\lambda_{4}}{dt}=-\frac{\partial ℋ}{\partial S_{ha}}=\left(\lambda_{2}-\lambda_{1}\right)\frac{b\beta_{m}S_{m}I_{h}}{N_{h}^{2}}+\left(\lambda_{5}-\lambda_{3}\right)\frac{b\beta_{hu}S_{hu}I_{m}}{N_{h}^{2}}\;\;\;\;\;\;+\left(\lambda_{4}-\lambda_{5}\right)\frac{\left(1-u_{3}\right)\left(b\beta_{ha}I_{m}N_{h}-b\beta_{ha}I_{m}S_{ha}\right)}{N_{h}^{2}}+\lambda_{4}\mu_{h},$$

$$\frac{d\lambda_{5}}{dt}=-\frac{\partial ℋ}{\partial I_{h}}=-A_{2}+\left(\lambda_{1}-\lambda_{2}\right)\frac{b\beta_{m}S_{m}N_{h}-b\beta_{m}S_{m}I_{h}}{N_{h}^{2}}+\left(\lambda_{5}-\lambda_{3}\right)\frac{b\beta_{hu}S_{hu}I_{m}}{N_{h}^{2}}\;\;\;\;\;\;+\left(\lambda_{5}-\lambda_{4}\right)\frac{\left(1-u_{3}\right)b\beta_{ha}S_{ha}I_{m}}{N_{h}^{2}}+\left(\lambda_{5}-\lambda_{6}\right)\varphi +\left(\lambda_{5}-\lambda_{7}\right)\gamma +\lambda_{5}\mu_{h},$$

$$\frac{d\lambda_{6}}{dt}=-\frac{\partial ℋ}{\partial P_{h}}\mathrm{\backslash textcolor}blue=-A_{3}+\left(\lambda_{2}-\lambda_{1}\right)\frac{b\beta_{m}S_{m}I_{h}}{N_{h}^{2}}+\left(\lambda_{5}-\lambda_{3}\right)\frac{b\beta_{hu}S_{hu}I_{m}}{N_{h}^{2}}\;\;\;\;\;\;+\left(\lambda_{5}-\lambda_{4}\right)\frac{\left(1-u_{3}\right)b\beta_{ha}S_{ha}I_{m}}{N_{h}^{2}}+\left(\lambda_{6}-\lambda_{7}\right)\varepsilon +\lambda_{6}\left(\mu_{h}+\delta \right),$$

$$\frac{d\lambda_{7}}{dt}=-\frac{\partial ℋ}{\partial R_{h}}=\left(\lambda_{2}-\lambda_{1}\right)\frac{b\beta_{m}S_{m}I_{h}}{N_{h}^{2}}+\left(\lambda_{5}-\lambda_{3}\right)\frac{b\beta_{hu}S_{hu}I_{m}}{N_{h}^{2}}\;\;\;\;\;\;+\left(\lambda_{5}-\lambda_{4}\right)\frac{\left(1-u_{3}\right)b\beta_{ha}S_{ha}I_{m}}{N_{h}^{2}}+\lambda_{7}\mu_{h},$$

with transversality boundary conditions $\lambda_{i}\left(t_{f}\right)=0$, i=1,2,…,7.

Next, taking the derivative of Hamiltonian function to control variables *u*_1_, *u*_2_, and *u*_3_, we obtain


$$\frac{\partial ℋ}{\partial u_{1}}=A_{4}u_{1}+\lambda_{1}\left(-\sigma S_{m}\right)+\lambda_{2}\left(-\sigma I_{m}\right),$$

$$\frac{\partial ℋ}{\partial u_{2}}=A_{5}u_{2}+\lambda_{3}\left(-\phi S_{hu}\right)+\lambda_{4}\left(\phi S_{hu}\right),$$ (16)

$$\frac{\partial ℋ}{\partial u_{3}}=A_{6}u_{3}+\lambda_{4}\left(\frac{b\beta_{ha}}{N_{h}}S_{ha}I_{m}\right)+\lambda_{5}\left(-\frac{b\beta_{ha}}{N_{h}}S_{ha}I_{m}\right).$$

Solving for *u*_1_, *u*_2_, and *u*_3_ from Eq (16) when $\frac{\partial ℋ}{\partial u_{1}}=0$, $\frac{\partial ℋ}{\partial u_{2}}=0$, and $\frac{\partial ℋ}{\partial u_{3}}=0$, we obtain


$$u_{1}=\frac{\sigma \left(\lambda_{1}S_{m}+\lambda_{2}I_{m}\right)}{A_{4}},$$

$$u_{2}=\frac{\phi S_{hu}\left(\lambda_{3}-\lambda_{4}\right)}{A_{5}},$$

$$u_{3}=\frac{b\beta_{ha}S_{ha}I_{m}\left(\lambda_{5}-\lambda_{4}\right)}{N_{h}A_{6}},$$

Taking the bounds of each control variables, we find the characterization of the optimal control as presented by


$$u_{1}^{*}=min\left(1,max\left(0,\frac{\sigma \left(\lambda_{1}S_{m}+\lambda_{2}I_{m}\right)}{A_{4}}\right)\;\right)\;,$$

$$u_{2}^{*}=min\left(1,max\left(0,\frac{\phi S_{hu}\left(\lambda_{3}-\lambda_{4}\right)}{A_{5}}\right)\;\right)\;,$$

$$u_{3}^{*}=min\left(1,max\left(0,\frac{b\beta_{ha}S_{ha}I_{m}\left(\lambda_{5}-\lambda_{4}\right)}{N_{h}A_{6}}\right)\;\right)\;.$$

## 7 Optimal control simulation

In this section, we discuss the numerical simulation of the optimal control problem using the forward-backward iterative method [39]. The parameter values used refer to Table 2 with the initial values are as follows:


$$S_{m}\left(0\right)=158,087,600,\;\;I_{m}\left(0\right)=300,\;\;S_{hu}\left(0\right)=26,347,933,$$

$$S_{ha}\left(0\right)=13,173,967,\;\;I_{h}\left(0\right)=2,000,\;\;P_{h}\left(0\right)=1,106,\;\;R_{h}\left(0\right)=100.$$

Next, we employ the values of weight constants as $A_{1}=A_{2}=A_{3}=1$, *A*_4_ = 20, *A*_5_ = 15, and *A*_6_ = 10. The parameter values of σ and ϕ are assumed to be σ=0.7 and ϕ=0.6. We consider seven control strategies, which is three for single intervention, three for double intervention, and one for full intervention.

**Fig 9:**
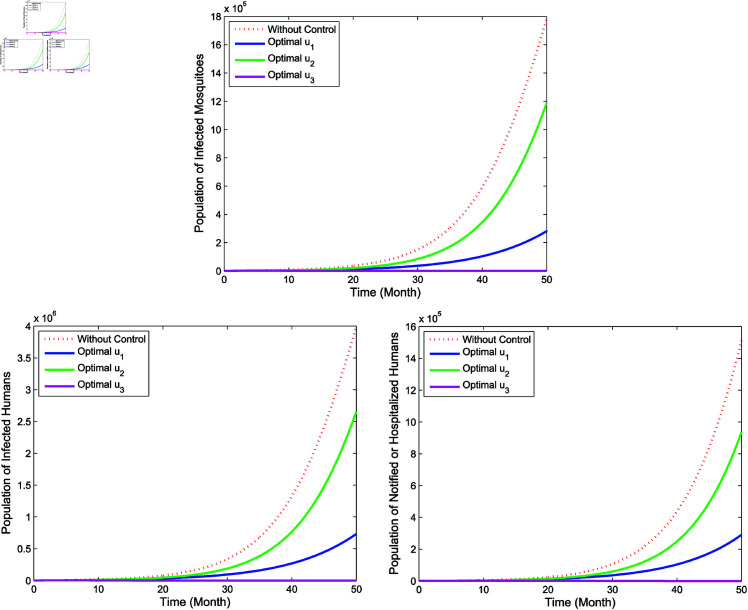
Simulation for optimal control for single intervention.

**Fig 10:**
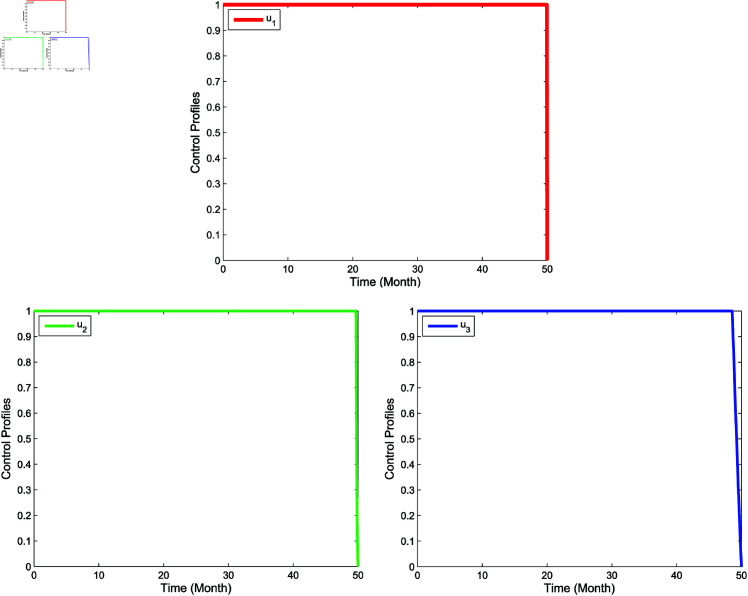
Control profiles for single intervention.

**Fig 11:**
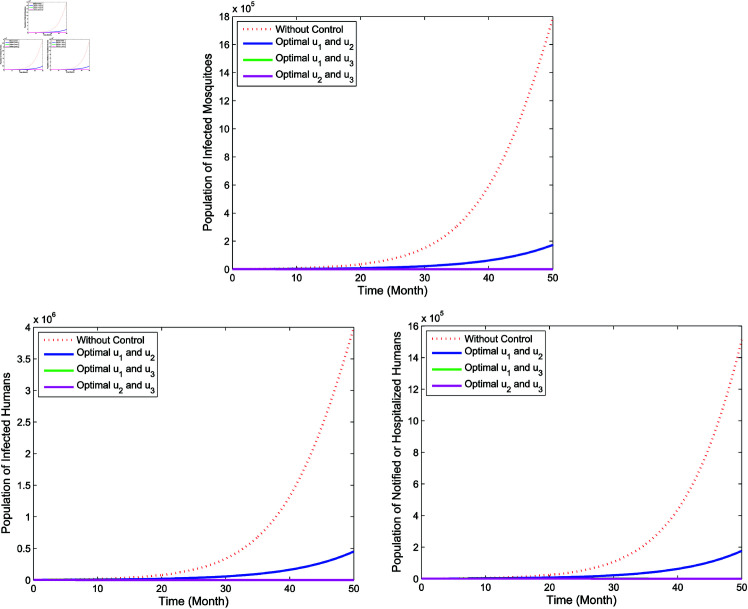
Simulation for optimal control for double intervention.

**Fig 12:**
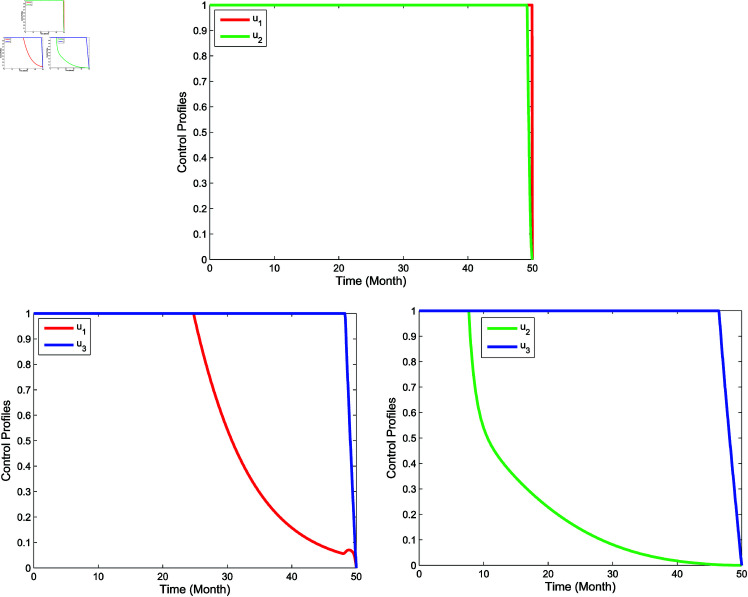
Control profiles for double intervention.

**Fig 13:**
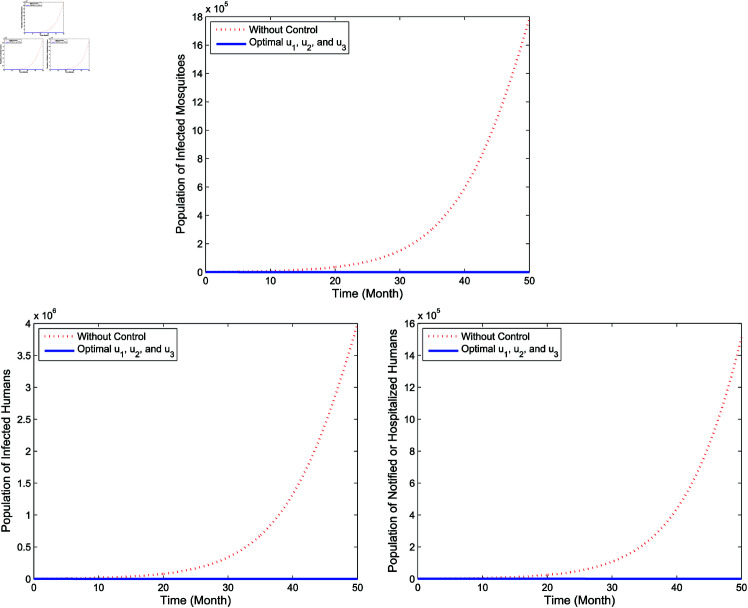
Simulation for optimal control for full intervention.

**Fig 14:**
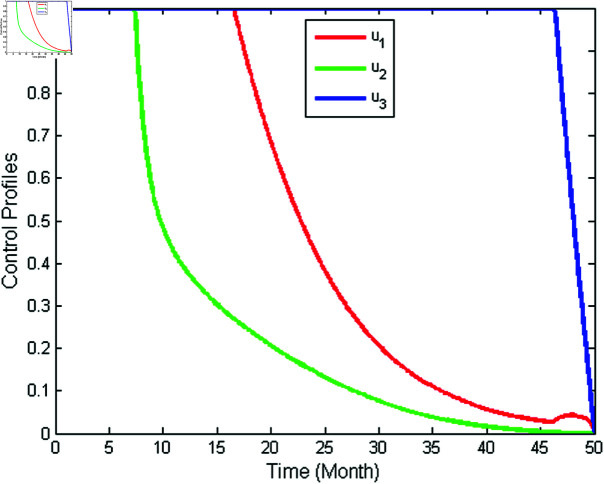
Control profile for full intervention.

1. **Single intervention**
  In single intervention, there are three control strategies, which is **Strategy A** (implementation of *u*_1_ only, while $u_{2}=u_{3}=0$), **Strategy B** (implementation of *u*_2_ only, while $u_{1}=u_{3}=0$), and **Strategy C** (implementation of *u*_3_ only, while $u_{1}=u_{2}=0$). The numerical results of the single intervention is illustrated in Fig 9 for compartments of *I*_*m*_, *I*_*h*_, and *P*_*h*_. Thus, the profile of the optimal control is shown in Fig 10. The simulation yield a lower populations of the infected mosquitoes (*I*_*m*_), the infected humans (*I*_*h*_) as well as the hospitalized and/or notified of infection (*P*_*h*_) when the single control measure is implemented compare to no control. Form Fig 10, it is apparent that the single control strategy for each of $u_{1},u_{2},$ and *u*_3_ should be carried out for a maximum of almost 50 months before it reduces to zero at the end of the period.
2. **Double intervention**
  In double intervention, we adopt three control strategies, namely **Strategy D**: the implementation of controls *u*_1_ and *u*_2_ $\left(u_{3}=0\right)$, **Strategy E**: the implementation of controls *u*_1_ and *u*_3_ $\left(u_{2}=0\right)$, and **Strategy F**: the implementation of controls *u*_2_ and *u*_3_ $\left(u_{1}=0\right)$. The simulation results in Fig 11 show that the double control intervention reduces significantly the number of the infected mosquitoes (*I*_*m*_), the infected humans (*I*_*h*_) as well as the hospitalized and/or notified of infection (*P*_*h*_) than the ones without the controls. The control profile of the double intervention is set out in Fig 12. For **Strategy D**, both controls *u*_1_ and *u*_2_ are given maximum until the end of the observation period in 50th month. Next, for **Strategy E**, the control *u*_1_ is given 100% for 25 months and then gradually reduced to zero at the end of the observation period, while control *u*_3_ is applied 100% for almost 50 months before finally being reduced to zero at the end of the observation period. Then, for **Strategy F**, the control *u*_2_ is initially set 100% for about 8 months and then gradually reduced to zero in the 50th month, while the control *u*_3_ is applied 100% for almost 48 months before finally going to zero at the end of the period.
3. **Full intervention**
  In full intervention, we apply **Strategy G**, which is the implementation of controls *u*_1_, *u*_2_, and *u*_3_ simultaneously. Fig 13 presents the implementation of full intervention for infected populations. As can be seen from Fig 13, Strategy G yields more significant reduction of the number of the infected mosquitoes (*I*_*m*_), the infected humans (*I*_*h*_) as well as the hospitalized and/or notified of infection (*P*_*h*_) than without the application of controls. The profiles of the three control variables simultaneously are illustrated in Fig 14. As shown in Fig 14, the control variable *u*_1_ is given a maximum for 16 months and reduced slowly to zero at the end of the period. Meanwhile, the control *u*_2_ is initially set at a maximum during 8 months then decreases gradually to zero at the end of the period. Next, the control *u*_3_ is supplied full effort during 46 months before decreases to zero by the conclusion of the period.

## 8 Cost effectiveness analysis

In this study, we perform a cost-effectiveness analysis to decide the most cost-effective strategy for applying the optimal control. To measure the disparity between the costs and health outcomes of the seven strategies, we employ the incremental cost effectiveness ratio (ICER) [40]. To avoid wasting limited resources, ICER is performed to compare two intervention strategies *i* and *j* with the formula as follows


$$ICER=\frac{Difference\;in\;total\;cost\;by\;strategies\;i\;and\;j}{Difference\;in\;the\;total\;number\;of\;averted\;infection\;by\;strategies\;i\;and\;j}.$$

The total number of averted infection is computed to be difference between the total number of infected and notified individuals without and with controls with the formula as follows


$$Total\;number\;of\;averted\;infection=\int_{0}^{t_{f}}\left(\left(I_{h}\left(t\right)-I_{h}^{*}\left(t\right)\right)+\left(P_{h}\left(t\right)-P_{h}^{*}\left(t\right)\right)\right)\;dt,$$

where the notation with subscript ^*^ is used to show the optimal solutions associated with the appropriate strategy. Meanwhile, the total cost generated by a control strategy refers to Eq (15). Note that for the ICER computation, at each step, the strategy with the highest ICER value is discarded or eliminated. When comparing 2 or more competing intervention strategies in a stepwise manner, one intervention is compared with the next-less-effective alternative in increasing order of total infected averted [40,41]. First, we arranged all of the strategy from smallest to highest number of total averted infection. Next, the ICER indexes is computed as follows


$$ICER\left(B\right)=\frac{3.7450\times 10^{7}-0}{1.8548\times 10^{7}-0}=2.02$$

$$ICER\left(A\right)=\frac{1.2716\times 10^{7}-3.7450\times 10^{7}}{3.6633\times 10^{7}-1.8548\times 10^{7}}=-1.37$$

$$ICER\left(D\right)=\frac{7.7247\times 10^{6}-1.2716\times 10^{7}}{4.0543\times 10^{7}-3.6633\times 10^{7}}=-1.28$$

$$ICER\left(C\right)=\frac{1.2363\times 10^{5}-7.7247\times 10^{6}}{4.6493\times 10^{7}-4.0543\times 10^{7}}=-1.28$$

$$ICER\left(E\right)=\frac{1.0413\times 10^{5}-1.2363\times 10^{5}}{4.6507\times 10^{7}-4.6493\times 10^{7}}=-1.39$$

$$ICER\left(F\right)=\frac{6.5521\times 10^{4}-1.0413\times 10^{5}}{4.6540\times 10^{7}-4.6507\times 10^{7}}=-1.17$$

$$ICER\left(G\right)=\frac{6.1894\times 10^{4}-6.5521\times 10^{4}}{4.6542\times 10^{7}-4.6540\times 10^{7}}=-1.81$$

Comparing Strategy B and Strategy A, the application of Strategy A is cost saving over Strategy B. This exhibit that the Strategy B is less effectiveness and more costly than the other strategy. Hence, Strategy B is discarded. Furthermore, we recalculation the index of ICER as follows


$$ICER\left(A\right)=\frac{1.2716\times 10^{7}-0}{3.6633\times 10^{7}-0}=0.35$$

$$ICER\left(D\right)=\frac{7.7247\times 10^{6}-1.2716\times 10^{7}}{4.0543\times 10^{7}-3.6633\times 10^{7}}=-1.28$$

$$ICER\left(C\right)=\frac{1.2363\times 10^{5}-7.7247\times 10^{6}}{4.6493\times 10^{7}-4.0543\times 10^{7}}=-1.28$$

$$ICER\left(E\right)=\frac{1.0413\times 10^{5}-1.2363\times 10^{5}}{4.6507\times 10^{7}-4.6493\times 10^{7}}=-1.39$$

$$ICER\left(F\right)=\frac{6.5521\times 10^{4}-1.0413\times 10^{5}}{4.6540\times 10^{7}-4.6507\times 10^{7}}=-1.17$$

$$ICER\left(G\right)=\frac{6.1894\times 10^{4}-6.5521\times 10^{4}}{4.6542\times 10^{7}-4.6540\times 10^{7}}=-1.81$$

Comparing Strategy A and Strategy D, the application of Strategy D is cost saving over Strategy A. This reveal the Strategy A is less effectiveness and more costly than the other strategy. Hence, Strategy A is wiped. Next, we recalculation the index of ICER as follows


$$ICER\left(D\right)=\frac{7.7247\times 10^{6}-0}{4.0543\times 10^{7}-0}=0.19$$

$$ICER\left(C\right)=\frac{1.2363\times 10^{5}-7.7247\times 10^{6}}{4.6493\times 10^{7}-4.0543\times 10^{7}}=-1.28$$

$$ICER\left(E\right)=\frac{1.0413\times 10^{5}-1.2363\times 10^{5}}{4.6507\times 10^{7}-4.6493\times 10^{7}}=-1.39$$

$$ICER\left(F\right)=\frac{6.5521\times 10^{4}-1.0413\times 10^{5}}{4.6540\times 10^{7}-4.6507\times 10^{7}}=-1.17$$

$$ICER\left(G\right)=\frac{6.1894\times 10^{4}-6.5521\times 10^{4}}{4.6542\times 10^{7}-4.6540\times 10^{7}}=-1.81$$

Comparing Strategy D and strategy C, the utilization of strategy C is cost saving over strategy D. This mean the strategy D is less effectiveness and more costly than the other strategy. Hence, strategy D is eliminated. Furthermore, we recalculation the index of ICER as follows


$$ICER\left(C\right)=\frac{1.2363\times 10^{5}-0}{4.6493\times 10^{7}-0}=0.003$$

$$ICER\left(E\right)=\frac{1.0413\times 10^{5}-1.2363\times 10^{5}}{4.6507\times 10^{7}-4.6493\times 10^{7}}=-1.39$$

$$ICER\left(F\right)=\frac{6.5521\times 10^{4}-1.0413\times 10^{5}}{4.6540\times 10^{7}-4.6507\times 10^{7}}=-1.17$$

$$ICER\left(G\right)=\frac{6.1894\times 10^{4}-6.5521\times 10^{4}}{4.6542\times 10^{7}-4.6540\times 10^{7}}=-1.81$$

Comparing Strategy C and Strategy E, the application of Strategy E is cost saving over Strategy C. This show that the Strategy C is less effectiveness and more costly than the other strategy. Hence, Strategy C is removed. Next, we recalculation the index of ICER as follows


$$ICER\left(E\right)=\frac{1.0413\times 10^{5}-0}{4.6507\times 10^{7}-0}=0.002$$

$$ICER\left(F\right)=\frac{6.5521\times 10^{4}-1.0413\times 10^{5}}{4.6540\times 10^{7}-4.6507\times 10^{7}}=-1.17$$

$$ICER\left(G\right)=\frac{6.1894\times 10^{4}-6.5521\times 10^{4}}{4.6542\times 10^{7}-4.6540\times 10^{7}}=-1.81$$

Comparing Strategy E and Strategy F, the utilization of Strategy F is cost saving over Strategy E. This indicate that the Strategy E is less effectiveness and more costly than the other strategy. Hence, Strategy E is removed. Next, we recalculation the index of ICER as follows


$$ICER\left(F\right)=\frac{6.5521\times 10^{4}-0}{4.6540\times 10^{7}-0}=0.001$$

$$ICER\left(G\right)=\frac{6.1894\times 10^{4}-6.5521\times 10^{4}}{4.6542\times 10^{7}-4.6540\times 10^{7}}=-1.81$$

When comparing Strategy F and Strategy G, the use of Strategy G is a cost savings over Strategy F. This means that Strategy F is less effective and more costly than the other strategy. Thus, Strategy F is eliminated. Our result suggests that Strategy G (full intervention) is the most cost-effective intervention associated with ICER. We present a summary of the ICER calculations in Table 4.

**Table 4:** Comparison of ICER for each intervention strategies.

Strategies	Optimal Control	Total Averted	Total Cost	ICER
B	$u_{2}^{*}$	$1.8548\times 10^{7}$	$3.7450\times 10^{7}$	2.02
A	$u_{1}^{*}$	$3.6633\times 10^{7}$	$1.2716\times 10^{7}$	–1.37
D	$u_{1}^{*}$ and $u_{2}^{*}$	$4.0543\times 10^{7}$	$7.7247\times 10^{6}$	–1.28
C	$u_{3}^{*}$	$4.6493\times 10^{7}$	$1.2363\times 10^{5}$	–1.28
E	$u_{1}^{*}$ and $u_{3}^{*}$	$4.6507\times 10^{7}$	$1.0413\times 10^{5}$	–1.39
F	$u_{2}^{*}$ and $u_{3}^{*}$	$4.6540\times 10^{7}$	$6.5521\times 10^{4}$	–1.17
G	$u_{1}^{*}$, $u_{2}^{*}$, and $u_{3}^{*}$	$4.6542\times 10^{7}$	$6.1894\times 10^{4}$	–1.81
Strategies	ICER-Recalculated-1	ICER-Recalculated-2	ICER-Recalculated-3	ICER-Recalculated-4
A	0.35	-	-	-
D	–1.28	0.19	-	-
C	–1.28	–1.28	0.003	-
E	–1.39	–1.39	–1.39	0.002
F	–1.17	–1.17	–1.17	–1.17
G	–1.81	–1.81	–1.81	–1.81
Strategies	ICER-Recalculated-5			
F	0.001			
G	–1.81			

## 9 Conclusion

This article studied the transmission dynamics and optimal control of dengue fever using a new mathematical model that takes into account both aware and unaware human populations. The results of the model analysis show that the stability of the disease-free equilibrium point is formed when *R*_0_<1, and it is further proven that the infection will persist in the population if *R*_0_ exceeds 1. This model was successfully applied to monthly data on dengue cases reported in East Java Province, Indonesia, during 2018–2020. In addition, we performed a sensitivity analysis to examine the dynamics of the dengue infection threshold and to identify the most sensitive factors that affect the incidence of dengue disease. The analysis shows that reducing the effective contact rate between susceptible and infected populations in both human and mosquito populations and increasing awareness programs are essential for the eradication of dengue. Next, by combining three control variables, a model with controls was built based on sensitivity analysis. The results of the control simulation demonstrated that each strategy was able to reduce the number of infections. In addition, a cost analysis of the optimal control problem shows that the combination of vector control, awareness programs, and awareness prevention is the most economically efficient approach.

The limitations of our study include the assumption that do not take into account age structure, no vaccination efforts, multi-strain infections are ignored, and there are no seasonal factors. Future research could focus on refining this model by incorporating additional factors, such as the implementation of vaccination campaigns and the influence of seasonal variations, which can significantly impact transmission patterns. Furthermore, it would be beneficial to explore the spatial dynamics of dengue spread, assess the role of human mobility, and consider heterogeneous population structures. These investigations could provide a more comprehensive understanding of dengue dynamics, leading to more effective and sustainable control strategies. In addition, collaborative efforts with local health agencies could improve data accuracy and ensure that the findings are translated into practical interventions for the improvement of public health. Future research can also explore different analytical methods to solve the model [42–45].

## Supporting information

S1 File Data. (PDF)
