# Supplementary material for: Modeling the Dynamics of Dengue Transmission with Awareness and Optimal Control Analysis
Source: PLoS One. 2025 May 22;20(5):e0322702. doi: 10.1371/journal.pone.0322702 (PMC12097629; doi:10.1371/journal.pone.0322702)
Supplement: S1 File — (PDF) [file pone.0322702.s001.pdf]

Data of Population Infected Human on 2018-2020 in East Java

Month (t)	Population Infected Human on the t month	Population Cumulative Infected Human on the t month
1	1106	1106
2	808	1914
3	759	2673
4	756	3429
5	749	4178
6	580	4758
7	523	5281
8	553	5834
9	586	6420
10	854	7274
11	964	8238
12	1214	9452
13	5587	15039
14	4220	19259
15	2739	21998
16	1604	23602
17	1360	24962
18	786	25748
19	443	26191
20	265	26456
21	245	26701
22	308	27009
23	397	27406
24	443	27849
25	1069	28918
26	1665	30583
27	1533	32116
28	1085	33201
29	870	34071
30	649	34720
31	402	35122
32	242	35364
33	229	35593
34	226	35819

35	279	36098
36	318	36416
